# The Influence of Ambient Factors on Emotional Wellbeing of Older Adults: A Review

**DOI:** 10.3390/s25041071

**Published:** 2025-02-11

**Authors:** Arturo Peralta, José A. Olivas, Francisco P. Romero, Pedro Navarro

**Affiliations:** 1 Escuela Superior de Ingeniería, Universidad Internacional de Valencia, Calle Pintor Sorolla, 21, 46002 Valencia, Spain; 2 Escuela Superior de Ingeniería y Tecnología, Universidad Internacional de La Rioja, Avda. de la Paz 93-103, 26006 Logroño, Spain; 3 Departamento de Tecnología y Sistemas de Información, Universidad de Castilla-La Mancha, Paseo de la Universidad, 4, 13071 Ciudad Real, Spain; 4 Tech Universidad Tecnológica, Av. Taco, 164, 38108 La Laguna, Spain

**Keywords:** designing for the elderly, environmental design, sensors, home health, information processing, knowledge acquisition

## Abstract

This work conducts a systematic review following PRISMA guidelines and using software tools like Covidence^®^ 2024 and Nvivo^®^ 15 for thematic analysis, aiming to examine significant studies on the impact of external factors on the emotional wellbeing of older adults and propose new conclusions and future research directions. In this context, studies using sensors to measure factors such as ambient temperature or lighting are key to understanding their impact on the emotional wellbeing of older individuals. These technologies offer opportunities to monitor and adapt environments in real-time, enabling targeted interventions. It is widely recognised that aspects like noise levels, ambient temperature, or lighting can influence an individual’s mood and overall wellbeing; however, it is crucial to further explore the effect of less studied factors. This review not only validates and questions popular beliefs about these factors but also highlights how the results can be useful for designing living environments that enhance the emotional wellbeing of the elderly and for establishing new directions in related research. By addressing these factors, this review provides actionable insights for policymakers, urban planners, and care providers to design environments that enhance the emotional wellbeing of older adults. Furthermore, this study not only validates previous knowledge but also highlights the need for future interdisciplinary interventions that integrate these factors holistically.

## 1. Introduction

It is now well recognised that when an individual performs a task, the ambient factors around them can influence the development and outcome of the activity. On occasions, the individuals may already be aware of these factors, and on others, they may be completely unforeseeable, making it even more complex to anticipate or mitigate the potential effects of the presence of such factors. The relationship between ambient factors and human wellbeing has been widely studied in environmental psychology, urban planning, and gerontology. Research has consistently shown that certain environmental conditions play a crucial role in shaping emotional and cognitive health. For older adults, these factors are particularly critical, as aging often brings increased vulnerability to environmental stressors, including sensory impairments, mobility limitations, and heightened physiological responses to temperature fluctuations.

Despite the growing recognition of these effects, much of the existing research has focused on younger and middle-aged populations, leaving a gap in understanding how ambient factors uniquely impact older adults. Furthermore, studies have traditionally examined individual environmental variables in isolation, rather than exploring their combined and interactive effects on wellbeing. Addressing these gaps is essential for developing comprehensive intervention strategies that can enhance the quality of life for aging populations, particularly in urban settings where environmental stressors are more pronounced.

Being aware of how environmental factors can affect the well-being of older adults is especially important and interesting in seeking to establish environments that enhance comfort, positive emotions, and a sensation of control or safety, key aspects in achieving greater physical and mental wellbeing, especially in older persons. The development and deployment of sensor-based technologies provide a unique opportunity to capture and analyse ambient factors in real-time. By leveraging data from environmental sensors, it becomes possible to design adaptive systems that respond to the specific needs of older adults, ensuring environments conducive to their emotional and physical wellbeing. For this reason, one of the primary research lines in applied psychology is the evaluation of the possible impact of certain factors on an individual’s mood or mental state, being an area of great interest for numerous researchers [1,2,3,4]. Nonetheless, the number of works focusing on groups of older adults is limited and there exists no review of the literature on this topic.

Thus, the aim of the current research is to review, analyse, and interpret the results of the studies in the field. To this end, we analyse both the works focused on the ambient factors traditionally addressed, such as temperature or lighting, and those that examine factors we consider to be important and which are not typically assessed, specifically engagement in activities that combine physical and mental activity, the presence of natural environments, or the influence of the social environment.

To guide this review, we address the following research question: “How do ambient factors influence the emotional wellbeing of older adults, and what implications do these findings have for designing environments that foster their psychological health?”. By answering this question, this review aims to synthesize existing evidence, identify research gaps, and propose strategies for integrating environmental considerations into urban planning, healthcare settings, and policy frameworks.

The knowledge gained will be useful to design living environments for older people that favour a greater sensation of emotional wellbeing as well as also to establish new lines of future research.

## 2. Materials and Methods

Between June 2024 and October 2024, a systematic review of the literature was conducted. For the exploratory search, we established the following MeSH (Medical Subject Headings) terms: Built Environment, Home Environment, Social Environments, Well Being, Health Status, Physical Health, Mental Health, Spiritual Well Being, Life Satisfaction. The types of studies considered were controlled clinical trials and observational studies, case series and case reports, all conducted with older adult populations, excluding bibliographic reviews and articles reporting experts’ subjective opinions. Additionally, other inclusion and exclusion criteria were established, depending on format or content, as defined in Table 1.

The procedure implemented to conduct the systematic review followed the protocols of the PRISMA declaration (Preferred Reporting Items for Systematic Review and Meta-Analysis) [5,6,7], and the analysis was performed using the Covidence^®^ platform [8]. Particular attention was given to studies that utilized sensor technologies to measure environmental factors such as noise levels, temperature, and lighting. These studies were included due to their potential to inform real-time interventions through objective and continuous data collection. The studies retrieved were independently assessed by expert reviewers for possible publication bias.

### 2.1. Data Collection

Before beginning the systematic review process, as a preliminary step, an initial search of studies related to the analysis of possible factors impacting the wellbeing of older adult populations was conducted in order to exclude terms that were found to be too broad and unnecessarily increased the sensitivity of the search. We were thus able to select more precise terms to enhance the specificity of the results. We selected the Scopus and PsycInfo databases for this search procedure.

As each database has its own thesaurus, each of the proposed search strings was adapted, combining indexed works and free terms. Table 2 shows the strategy used in PsycInfo.

Finally, after selecting the most appropriate keywords, the strategy implemented in the definite search strategy was as follows: (“Physical Surroundings” OR “Living Conditions” OR “Family Environment”) AND (“Wellness” OR “Welfare”) OR (“Health Condition” OR “Physical State” OR “Physicals Conditions”) OR (“Psychic Well-Being” OR “Personal Satisfaction” OR “Emotional Stability”).

**Figure 1:**
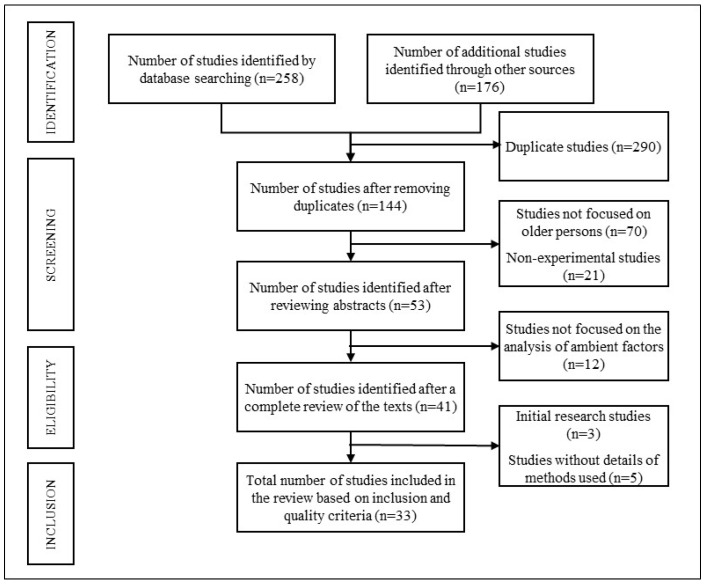
Flow diagram for the study selection process.

In addition to conducting the searches in the selected databases (Scopus and PsycInfo), we included the results of manually conducted searches of the foremost journals in the field (Figure 1). Additionally, an analysis of the grey literature (material not indexed by major databases) related to the area was conducted by consulting the opengrey.eu website.

After the search was completed, the results were exported to Covidence^®^, a tool designed to support the process of evaluating the studies retrieved through a systematic review.

Following the phases defined in Figure 1, the first step was to eliminate any duplicate publications. Then, we read the title and abstract in detail, excluding those that failed to match the topic matter under study. Finally, we reviewed the complete text of each publication, eliminating works that were found to be irrelevant according to our inclusion and exclusion criteria. Additionally, the process to select and exclude publications was assisted by the presence of two expert reviewers in the context, and a third reviewer, whose intervention was eventually unnecessary due to the high value of inter-rater reliability obtained, higher than 0.9 according to Cohen’s kappa coefficient.

Especially important for the final selection of the qualitative, quantitative, and mixed studies was the assessment of their quality, for which we followed the protocol of “Questions to ask of research or evaluation evidence” from BEME Guide No. 1 [9]. This yielded the final set of studies, and the data analysis phase could then begin.

### 2.2. Data Analysis

In order to detect recurrent topics in the set of qualitative, quantitative, and mixed studies that could be summarised and catalogued under thematic headings, we used the Nvivo^®^ 15 software [10,11] for the process of extracting and synthesising information. To ensure a systematic and structured analysis, we used Nvivo^®^ 15 software, which facilitates the organization, coding, and synthesis of large volumes of qualitative data. Compared to manual reading, Nvivo^®^ offers greater consistency and efficiency, reducing the risk of human bias and enabling the identification of patterns and co-occurrences across multiple studies. One key advantage of Nvivo^®^ is its ability to quantify qualitative trends, providing insights into the prevalence of specific ambient factors. However, its reliability depends on the quality of the initial coding framework. To mitigate this, we developed a predefined thematic structure based on prior literature and refined it iteratively through manual validation. While alternative software solutions such as ATLAS.ti and MAXQDA exist, we selected Nvivo^®^ due to its robust analytical capabilities and visualization tools, which allowed for a deeper exploration of relationships between environmental factors and emotional wellbeing. By combining Nvivo-assisted analysis with researcher validation, we ensured that the extrapolations were both accurate and contextually meaningful.

As part of this process, we categorized environmental factors as “influential” or “non-influential” based on their reported impact on emotional wellbeing in the reviewed studies. This classification does not imply statistical significance, as not all studies performed quantitative hypothesis testing. Instead, a factor was considered influential if multiple studies consistently reported qualitative or quantitative evidence of its effect, even in the absence of formal statistical validation. Conversely, a factor was classified as noninfluential if the reviewed studies provided either weak or inconclusive evidence regarding its impact. This distinction is essential to prevent misinterpretation, particularly in contrast with statistical significance, which applies only to studies with hypothesis-driven analyses. Our classification is based on a qualitative synthesis of the existing literature, ensuring that the findings reflect the weight of reported evidence rather than a purely statistical evaluation.

With the classification framework established, the analysis proceeded in three phases.

The aim of the first phase was to synthesise all the information obtained from the final selection of the 33 relevant retrieved studies. To this end, an open coding process was conducted, whereby the different categories related to each of the studies were recorded.

In the second phase, the categories were described and organised by topic, depending on the what, why, and how of the analysis of the factors influencing the wellbeing of older persons.

Finally, in the third phase, an interpretative process was conducted, with the aim of generating a summary report of the information obtained from each study and its conclusions. This final report was elaborated following the STORIS protocol (structured approach to the reporting in healthcare education of evidence synthesis) [12].

## 3. Results

Following the process depicted in Figure 1, the initial search of the databases gave rise to 258 records. Additionally, through searches of other sources, 176 articles were retrieved, making a total of 434 studies. After eliminating duplicate studies and those with little relationship to the topic, we excluded a total of 290 records, retaining 144 articles considered relevant.

Subsequently, the content of each of the 144 selected studies was reviewed. First, we reviewed the abstracts, reducing the number of relevant studies to 53. The complete manuscripts were then reviewed, resulting in a list of 41 publications. A final additional review was conducted, applying our inclusion and exclusion criteria, generating a set of 33 selected articles. Table 3 lists the most significant characteristics of each study.

All the studies were conducted between 2009 and 2024, with more than half of them being performed in the last three years.

All the studies retrieved were experimental, involving a total of 177,888 participants. Additionally, the clinical records of 22,562 deceased patients were analysed [34], and data on 365,296 hospital admissions or visits for mental health problems were processed [40,41].

Of the 177,888 participants involved in the selected studies, 34,984 had a recorded mental health diagnosis [17,18,25,31,34,37,38,39].

### 3.1. Why Is the Effect of Ambient Factors on Older Persons’ Wellbeing Analysed?

The essential, common aim of the works extracted from the databases was to analyse the impact of certain factors on the health of older persons. In eight of the studies, this effect was examined in conjunction with aspects related to physical and mental health [15,16,19,23,32,33,34,37], while, in the remaining 25, the effect was analysed only in terms of wellbeing or mental health [13,14,17,18,20,21,22,24,25,26,27,28,29,30,31,35,36,38,39,40,41,42,43,44,45].

Furthermore, in a subset of seven works, the experimental phase was designed with the aim of comparing the results across age groups [13,15,16,32,38,40,41]. A group of four studies included participants with different types of illnesses to specifically evaluate the level of the effects in patients with physical [30,43] or mental pathologies [17,18,25,31,34,37,38,39].

### 3.2. What Ambient Factors Are Analysed as Possibly Having an Impact on Older Persons’ Wellbeing?

Eight types of factors are analysed in the selected studies. Specifically, the impact of music, through interventions based on music therapy, is analysed in four works [13,24,30,43], noise is examined in eleven [15,16,18,19,22,23,31,32,33,35,44], climate and aspects of temperature and weather are analysed in thirteen [14,15,16,17,20,25,28,34,36,37,38,40,41], the social environment is addressed in seven [21,22,23,26,42,44,45], the closeness of green spaces is analysed in four [27,29,33,44], the air quality in four [15,16,37,44], the lighting conditions in two [15,16], and the stress in one [39].

It is worth noting that all the studies focused on the analysis of a single factor, with the exception of the following works: those by [22,23], which analysed the effect of the social environment and noise in conjunction; the study by [33], which investigated the effect of the presence of greenness and noise; Refs. [15,16] which jointly analysed temperature, noise levels, lighting conditions, and air quality in individuals with autism, ref. [37] which examined the combined effects of temperature and air quality in dementia care environments, and [44] which analyses architectural design, medical care, and environmental factors like social environment, greenness, noise, and air quality.

In particular, a subset of studies incorporated sensor-based methodologies to measure ambient factors like noise levels, temperature, and lighting conditions. These sensors enabled precise, continuous data collection, ensuring more reliable results compared to subjective reporting. For example, noise levels were captured using calibrated sound level meters, while lighting and temperature variations were tracked with photometric and thermal sensors, respectively. These tools allowed researchers to evaluate the direct impact of environmental changes on emotional wellbeing and uncover patterns that might otherwise go unnoticed through traditional methods.

### 3.3. How Is the Influence of Ambient Factors on Older Persons’ Wellbeing Analysed?

A variety of techniques are used to collect the data on which to apply statistical or, in general, mathematical models to analyse the impact of the selected factors on health status. Surveys and standardised assessments remain a prevalent approach. Ten studies relied on questionnaires to subjectively assess participants’ perceived emotional wellbeing [13,20,21,23,28,39,42,43,44,45], or standardised cognitive tests to measure cognitive capacity [30], serving as the foundation for their analyses and conclusions. Similarly, ref. [16] employed a survey-based methodology to examine how age, gender, and comorbidities influence environmental sensitivity, incorporating statistical techniques to distinguish trends across subgroups.

Other studies apply statistical methods grounded in correlation and regression models to assess the association between environmental factors and health-related events, such as the effect of weather conditions on hospital visits for mental health issues [14,40,41] or the daily mortality rate [34]. Additionally, mathematical models have been employed to evaluate the relationship between ambient noise and sleep quality [32], general health status [19,35], and suicide incidence [31], as well as the correlation and potential causality between green spaces and subjective wellbeing [27,29].

In contrast, expert knowledge and objective criteria are leveraged in some studies to assess physical and cognitive capacity, complementing statistical models [22,24,26,33]. In [37,38] utilised qualitative research methods, including semi-structured interviews with caregivers, to explore how temperature and air quality affect older adults with dementia in residential settings. Similarly, ref. [25] conducted interviews with older adults in dementia-friendly housing to assess their thermal comfort perceptions, emphasising autonomy over climate control as a key factor in emotional wellbeing.

Among the methods, studies employing sensor technologies stood out for their ability to provide objective and granular data. For instance, ref. [17] utilised infrared thermography to evaluate differences in skin temperature and thermal sensation between older adults with and without dementia, demonstrating physiological discrepancies that subjective reports alone might not reveal. Likewise, ref. [18] applied acoustic modelling techniques to predict noise levels in nursing homes and assess their potential impact on residents with dementia. In [15], a sensor-based environmental monitoring was integrated to evaluate temperature, noise, lighting, and air quality in individuals with autism, reinforcing the role of multifactorial environmental analysis. Additionally, ref. [36] conducted a field study in six nursing homes, combining long-term indoor climate monitoring with thermal comfort surveys.

These advanced tools not only enriched the dataset but also facilitated cross-sectional and longitudinal comparisons, offering new insights into how specific ambient factors directly correlate with emotional wellbeing in older adults. The integration of quantitative modelling, expert assessments, and sensor-based methodologies presents a comprehensive approach for studying the complex interactions between environmental conditions and health outcomes in ageing populations.

### 3.4. What Are the Findings on the Impact of the Factors Studied?

Table 4 shows the findings of the selected studies, grouped together according to the factor analysed and showing the influence identified, or not, at a physical and mental level. It is worth noting that some studies analyse various factors, while not all the works address the possible impact on physical health, as the focus of the selection of studies was the assessment of the impact of ambient factors on mental wellbeing.

First, worth noting is that the four studies analysing the impact of music therapy all conclude it has a positive effect on psychological wellbeing but present no experiments or results on the possible impact on physical health.

Similarly, the six studies on the impact of the social environment agree on its robust relationship with perceived wellbeing, without making any assessment of its importance in physical wellbeing. Additionally, all the works on the significance of noise underline its impact on both physical and mental health. Although only one study analyses the influence of stress, it clearly confirms its significance for emotional wellbeing.

As regards the impact of temperature and weather conditions, twelve of the thirteen works analysing this factor find an association between weather and mental wellbeing, while the remaining study reports no such relationship. Finally, although fewer studies were retrieved on the impact of green spaces, stress, lighting, and air quality, all of them reported their impact on mental wellbeing but did not present significant conclusions regarding physical health aspects.

This analysis underscores that no single environmental factor acts in isolation. Instead, their effects appear to be interdependent, suggesting that future interventions should consider multi-factorial approaches rather than addressing each element in isolation. These insights strengthen the argument for a holistic design framework aimed at fostering healthier living environments for older adults.

The reviewed studies consistently highlight the significance of specific ambient factors in shaping the emotional wellbeing of older adults. Noise levels, for instance, were frequently associated with both increased stress and cognitive fatigue, whereas green spaces were linked to reduced anxiety and enhanced mood stability. The social environment emerged as a pivotal factor, influencing both positive emotional states and overall life satisfaction. Moreover, air quality and ventilation were recurrently mentioned as critical elements, particularly in institutional settings, where poor conditions were correlated with higher distress levels and reduced comfort.

In summary, the analysis highlights the consistent impact of noise, the configuration of a well-designed musical environment, climatic conditions, the presence of green spaces, stress, lighting, air quality, and social environments on mental wellbeing, while also identifying gaps in research related to physical health outcomes and the analysis of combined factors.

## 4. Discussion

The analysis of the impact of ambient factors on older persons’ wellbeing is currently a research area of significant interest, given that all the studies extracted were published after 2009, and eight between 2021 and 2024. The growing interest in this area aligns with the accelerated ageing of the global population, which presents unique challenges for creating environments that foster emotional wellbeing. Understanding how ambient factors influence older adults has not only scientific implications but also practical ones, such as informing public policy and developing targeted intervention strategies. Building on this growing interest, the studies reviewed exemplify the increasing focus on understanding the nuanced ways ambient factors shape wellbeing and provide critical insights into their psychological and physical impacts on older adults.

The reviewed studies highlight that interventions such as improved ventilation, noise reduction strategies, and access to natural environments can significantly enhance older adults’ emotional wellbeing. In practice, this means that urban planning should prioritise pedestrian-friendly, quieter zones with integrated greenery, while healthcare settings should leverage sensor-based monitoring to adjust indoor conditions in real time. These targeted interventions can help mitigate environmental stressors and promote sustainable, health-oriented design solutions for ageing populations.

As regards the target of the selected studies, although only 8 of the 33 works analyse both the impact on physical and mental wellbeing, it should be noted that the aim of the present review was primarily to retrieve research on the psychological influence. Thus, it is reasonable to expect a smaller number of studies assessing both aspects, and hence, we are unable to conclude which perspective, mental or physical wellbeing, generates greater research interest. This limitation presents an opportunity for future research to adopt a more holistic approach by integrating both physical and mental wellbeing outcomes. Examining these domains in conjunction would provide a more comprehensive understanding of how ambient factors influence older adults’ overall quality of life. For instance, chronic exposure to environmental noise has been shown to not only induce stress and anxiety but also contribute to cardiovascular and metabolic disorders. Similarly, inadequate lighting may negatively impact circadian rhythms, leading to disruptions in both sleep and mood regulation. By analysing these interactions, researchers can better understand the bidirectional relationships between physical and mental health and identify potential mediating variables. However, implementing such an integrative approach presents methodological and practical challenges. First, it requires multidisciplinary methodologies, incorporating physiological health assessments (e.g., biomarkers, sleep quality metrics, cardiovascular indicators) alongside psychological evaluations (e.g., mood scales, cognitive function tests). This necessitates collaboration across fields such as gerontology, psychology, medicine, and environmental science. Additionally, longitudinal study designs may be needed to track the long-term interactions between ambient factors, physical health deterioration, and mental wellbeing changes over time. Future studies could benefit from holistic approaches that examine how these domains interact, particularly in the context of ageing, where physical limitations often have cascading effects on emotional health. For instance, chronic exposure to environmental noise has been shown to exacerbate stress responses, potentially triggering both physical and psychological health declines. Older adults may be particularly susceptible to such effects due to age-related sensory and cognitive changes. Understanding these vulnerabilities highlights the importance of designing interventions that mitigate such environmental stressors. For instance, interventions could include soundproofing measures in care homes or promoting quieter community spaces to reduce stress and improve quality of life.

As regards the methods used, 32% of the studies administered questionnaires designed by the authors, while 7% employed standardised scales to measure capacities, and 13% relied on expert evaluations. The remaining studies focused on statistical analyses of specific events, such as hospitalisations, mortality rates, or the measurement of environmental parameters like noise or temperature levels. Consequently, nearly half of the studies (52%) were based on participants’ self-assessments through various questionnaires, while the remaining 48% relied on objective criteria, such as expert assessments or causal event analysis.

Self-assessments provide valuable insights into subjective experiences, but their reliance on individual perception introduces potential biases, including memory distortion and social desirability effects. To address these limitations, future research should adopt hybrid methodologies that integrate self-reports with objective physiological and behavioural data. Experience Sampling Methods (ESM), for instance, allow participants to provide real-time emotional state reports, reducing recall bias while maintaining subjective nuance. Additionally, passive data collection using wearable sensors—such as heart rate variability monitors, accelerometers, and sleep trackers—can offer physiological indicators that complement self-reported measures of emotional wellbeing.

A significant portion of the studies reviewed leveraged sensor technologies to enhance objectivity. These technologies enable the continuous and real-time monitoring of environmental and physiological variables, offering granular insights into how ambient conditions impact wellbeing. For example, calibrated sound level meters have provided precise measurements of noise exposure, photometric sensors have quantified lighting conditions, and thermal sensors have tracked temperature fluctuations. Moreover, AI-driven analytics allow for the identification of subtle patterns within these datasets, refining the correlation between environmental factors and emotional responses. Despite these advantages, implementing sensor-based research with older adults necessitates careful ethical considerations. Issues such as informed consent, data privacy, and potential discomfort with continuous monitoring must be addressed. Researchers should ensure clear and transparent communication about data collection procedures, emphasising how information will be used and securely stored. Employing robust anonymisation protocols and encryption techniques can help safeguard participants’ privacy while maintaining data integrity. Additionally, tailoring consent processes to the cognitive and technological literacy levels of older adults will enhance participant understanding and engagement. By balancing technological advancements with ethical responsibility, future research can maximise the benefits of sensor-based methodologies while preserving the autonomy and dignity of older individuals. The integration of advanced technologies, including big data analytics and AI-enhanced environmental monitoring, could further enrich the reliability of findings. Such methodological advancements will not only mitigate self-reporting biases but also pave the way for scalable and personalised interventions that optimise living environments for ageing populations.

Regarding the factors analysed and the results obtained, it is significant that weather, noise, and the social environment are by some distance the most widely studied elements and are all robustly associated with emotional wellbeing, although the works provide no clear conclusions on their level of effect on physical wellbeing due to a lack of research focus on this perspective. The influence of weather, noise, and social environment on emotional wellbeing in older adults can be explained through distinct psychological and physiological mechanisms. Weather conditions, particularly temperature extremes, have been linked to mood fluctuations due to their effects on thermoregulation and physiological stress responses. High temperatures can exacerbate discomfort, dehydration, and cardiovascular strain, which in turn may heighten anxiety and irritability. Conversely, exposure to natural light has been associated with improved mood regulation through its role in circadian rhythm alignment and serotonin production. Environmental noise primarily affects emotional health through its impact on stress and cognitive load. Chronic exposure to high noise levels has been shown to activate the hypothalamic-pituitary-adrenal (HPA) axis, leading to elevated cortisol levels and an increased risk of anxiety and depressive symptoms. Noise pollution also disrupts sleep quality, which is a critical factor for emotional stability, particularly in older adults who are more susceptible to sleep disturbances. Additionally, cognitive decline may reduce the ability to filter out background noise, making older individuals more vulnerable to the negative effects of persistent environmental noise. The social environment plays a dual role, acting as both a protective and a risk factor for emotional wellbeing. Strong social networks and frequent social engagement have been linked to lower rates of depression and anxiety, as they provide emotional support, cognitive stimulation, and a sense of belonging. On the other hand, social isolation and loneliness are associated with increased stress, higher levels of systemic inflammation, and an elevated risk of cognitive decline, highlighting the importance of social connectivity in later life. Moreover, the effects of these ambient factors are not uniform across all older adults. Subgroups such as individuals with pre-existing mental health conditions, those with sensory impairments (e.g., hearing loss), and those living in urban environments may experience heightened sensitivity to noise and environmental stressors. For example, older adults with cognitive decline may struggle more with noise pollution, as it can exacerbate confusion and agitation. Similarly, those with mobility issues may be more affected by extreme weather conditions due to limited access to temperature-controlled environments. Socioeconomic status also plays a role, as individuals in lower-income communities may have reduced access to quiet living spaces, green environments, and social engagement opportunities. Recognising these subgroup variations is crucial for designing targeted interventions. Urban planning strategies, such as creating quiet zones, integrating green spaces, and improving community infrastructure, can help mitigate environmental stressors. Additionally, personalised interventions, such as soundproofing solutions for individuals with hearing loss or structured social programs for isolated older adults, could enhance emotional resilience in specific populations. Future research should explore how multi-factorial interventions—combining environmental modifications, social support, and adaptive technologies—can more effectively promote emotional wellbeing across diverse older adult populations.

The only study that argues against weather having an impact on emotional wellbeing is that by [28], which is an especially significant work given the size of the sample, namely a million inhabitants of the United States over five years. Nonetheless, as underlined by the authors themselves when describing the limitations of their study, despite the conclusions being robust, studies have shown that the effects of climate can be substantially affected by moderator variables when making life satisfaction judgements. For example, ref. [46] reported gender differences in the links between weather and life satisfaction, while [47] reported significant random variation across individuals as regards the effects of weather. Thus, in the study by [28], we cannot rule out the possibility of weather having substantial effects on significant subgroups, such as older persons, within our sample. A promising direction for future research lies in exploring the interactions between multiple ambient factors. For instance, how environmental noise might moderate the positive effects of green spaces. Additionally, it is essential to consider cultural and geographical differences, as perceptions of the environment and its impact can vary significantly across regions. Ensuring the representativity of diverse populations in future research requires methodological adjustments, including cross-cultural comparative studies, the use of region-specific datasets, and the incorporation of community-driven research approaches. Collaborating with local researchers and stakeholders can help design studies that account for culturally specific perceptions of environmental factors. Additionally, employing multi-site research designs across different geographical contexts can provide a broader understanding of how ambient factors influence wellbeing in varied settings. Cultural and geographical differences also play a crucial role in shaping the effectiveness of environmental interventions. For instance, while green spaces have been shown to improve emotional wellbeing, their impact may be moderated by cultural attitudes towards nature or the availability of public recreational areas. Similarly, social environments may have differing effects depending on societal norms surrounding ageing and community engagement. Recognising these variations is essential for tailoring interventions that are both effective and contextually relevant, ensuring that strategies such as urban planning, noise reduction measures, and social programs align with the specific needs and expectations of older adults in diverse populations. Understanding these interactions could guide the development of multifactorial interventions, such as combining noise reduction strategies with green space enhancement, to maximise benefits for emotional wellbeing. For instance, urban designs incorporating sound barriers alongside green spaces may amplify their individual benefits, creating restorative environments for older adults. Such designs would not only benefit older adults but also contribute to healthier, more inclusive urban environments for all age groups.

Finally, although the number of studies retrieved is smaller than for the previous factors, there is a clear consensus on the association between stress, lighting conditions, air quality, greenness, and music therapy and individuals’ psychological wellbeing. The findings on these factors highlight the need to consider these elements in the design of environments tailored for older adults. For example, personalised music therapy programmes in care homes or accessible green spaces in urban areas could serve as effective strategies to enhance the emotional wellbeing of this population. These findings underscore the importance of interdisciplinary collaboration to develop practical solutions that bridge the gap between research and real-world applications, ultimately improving the quality of life for older adults. To foster effective interdisciplinary collaboration, structured frameworks such as living labs, joint research initiatives, and policy-driven partnerships should be implemented. Living labs bring together researchers, urban planners, healthcare professionals, and technologists to co-design and test environmental interventions in real-world settings. Similarly, joint research initiatives can be promoted through funding agencies and academic-industry partnerships, encouraging cross-disciplinary studies that integrate environmental design, psychological wellbeing, and healthcare innovations. Policy-driven collaborations with municipal governments and urban developers can help translate research findings into actionable public policies. The insights from these collaborations can be translated into practical solutions by designing restorative environments tailored to older adults’ needs. For instance, integrating sensor-based adaptive environments—where real-time monitoring of noise, lighting, and air quality informs automated adjustments—can enhance comfort and emotional wellbeing. Additionally, incorporating biophilic urban design, such as increased green spaces and therapeutic gardens, can promote relaxation and cognitive health. Community-based interventions, including structured social engagement programs and mobility-friendly urban planning, can further enhance the psychological resilience of older adults. By leverageing interdisciplinary expertise, future research can move beyond theoretical discussions to implementable solutions that improve the everyday experiences of ageing populations. Further longitudinal and large-scale studies are essential to validate these findings and refine interventions for diverse older populations. This calls for collaboration among urban planners, environmental psychologists, geriatricians, and technologists to ensure comprehensive and impactful solutions.

The findings of this review offer a clear response to the research question, confirming that ambient factors such as noise, weather, and social environment significantly shape the emotional wellbeing of older adults. However, the level of impact varies depending on individual vulnerabilities, cultural contexts, and the degree of environmental exposure. From an applicability perspective, these insights can be translated into specific recommendations:

- For urban planning: prioritizing noise reduction, increasing access to green spaces, and designing environments that promote social interaction.
- For healthcare and assisted living facilities: implementing sensor-based monitoring systems that allow for adaptive environmental adjustments to optimise wellbeing.
- For policymakers: establishing regulations that ensure age-friendly environmental standards in residential areas and public spaces.

These results emphasise the need for an interdisciplinary approach that integrates urban planning, healthcare innovation, and psychological research to develop evidence-based interventions that enhance the emotional resilience of older populations.

## 5. Conclusions and Future Work

This review highlights the fundamental role of ambient factors in the emotional wellbeing of older adults, emphasising their influence on quality of life and mental health. It confirms that elements such as noise, climatic conditions, the social environment, and music therapy have a significant impact on emotional wellbeing, although the magnitude of this effect varies depending on individual vulnerabilities, cultural contexts, and levels of environmental exposure. However, their relationship with physical health remains a less explored area, leaving open questions regarding the potential cumulative effects of prolonged exposure to certain environmental factors on the physical condition of older adults.

From an application perspective, these findings can be translated into concrete interventions across various domains. In urban planning, reducing noise levels, increasing access to green spaces, and designing environments that promote social interaction may enhance the wellbeing of this population. In healthcare and care institutions, the implementation of sensor-based environmental monitoring systems would allow real-time adjustments to optimise environmental conditions, while strategies such as music therapy could be integrated into psychological wellbeing programmes to reduce anxiety and improve emotional regulation. Furthermore, from a public policy perspective, it is crucial to develop regulations that ensure age-friendly environmental standards in residential areas and public spaces, fostering settings that minimise environmental stressors and promote emotional stability.

Despite advances in research on these topics, multiple questions remain that require further exploration in future studies. Firstly, most studies have addressed environmental factors in isolation, without considering their interactions. It is essential to investigate how the combination of factors, such as the joint influence of noise and greenery in stress reduction or the relationship between lighting and circadian rhythm regulation, may amplify or mitigate their effects on health. Additionally, while the link between environmental factors and mental wellbeing is well documented, their relationship with physical health remains an open question. Longitudinal studies analysing the long-term impact of these factors on cardiovascular, metabolic, or neurological health in older adults would provide a deeper understanding of their cumulative effects.

While previous studies have typically examined ambient factors in isolation, future research should explore how multiple factors interact to affect emotional wellbeing. For example, analysing the combined effects of noise reduction and green space exposure could offer valuable insights into how urban design can mitigate environmental stressors holistically. Similarly, investigating the interplay between lighting conditions and circadian rhythm regulation could enhance strategies for improving sleep quality and emotional stability in older adults. By incorporating multidisciplinary methodologies—including sensor-based monitoring, longitudinal studies, and machine learning analytics—future research can uncover hidden patterns and optimise interventions for ageing populations.

Another key aspect for future research is the evaluation of mitigation strategies. Although various interventions have been proposed, such as incorporating biophilic design in urban environments or applying music therapy programmes in care homes, empirical validation of their effectiveness across different contexts is necessary. Similarly, advances in smart technologies and the use of sensors to assess environmental parameters in real-time offer new opportunities to design adaptive environments that optimise temperature, noise, or lighting according to the specific needs of older adults. The application of big data analytics and machine learning in this field could revolutionise the way spaces are designed for healthy ageing.

Finally, it is essential to expand the representativeness of studies, as most reviewed research has been conducted in Western contexts, leaving unexplored how environmental factors affect older adults in different cultural and socio-economic settings. The inclusion of more diverse populations would allow for the development of more inclusive and tailored intervention strategies.

Overall, these findings provide a solid framework for future research at the intersection of environmental psychology and the wellbeing of older adults. The combination of traditional methodologies with emerging technological approaches will enable the design of more precise and effective interventions, moving towards the creation of environments that not only minimise the negative impacts of environmental factors but also actively enhance wellbeing and quality of life in an ageing population.

## Figures and Tables

**Table 1:** Inclusion and exclusion criteria.

Inclusion Criteria	Exclusion Criteria
Studies analysing the influence of ambient factors on the wellbeing of older adults over 65 years old.	Research focused on the study of the influence of nonenvironmental factors on health.
Studies on the mental health status or wellbeing of older adults, where the environmental factors possibly responsible for their health status are analysed.	Studies focused on the analysis of older persons’ health or wellbeing where the analysis is conducted without sufficient data on related factors or lifestyles.
Empirical or primary studies.	Systematic reviews and opinion articles, such as editorials, letters to the editor, comments or points of view.
Quantitative or qualitative studies with appropriate definitions, data analyses, and valid conclusions.	Quantitative or qualitative studies without precise findings.
Studies written in Spanish or English.	Studies not written in Spanish or English.

**Table 2:** Final search strategy used in PsycInfo.

Search Strings
Search 1 (terms related to OR): MeSH terms: Built Environment, Home Environment, Social Environments Free terms: Physical Surroundings, Living Conditions, Family Environment
Search 2 (terms related to OR): MeSH terms: Well Being Free terms: Wellness, Welfare
Search 3 (terms related to OR): MeSH terms: Health Status, Physical Health Free terms: Health Condition, Physical State, Physicals Conditions
Search 4 (terms related to OR): MeSH terms: Mental Health, Spiritual Well Being, Life Satisfaction Free terms: Psychic Well-Being, Personal Satisfaction, Emotional Stability
Search 5: Search 1 AND (Search 2 OR Search 3 OR Search 4)

**Table 3:** Main characteristics of the selected studies.

Study	Aims (Why?)	Factors (What?)	Methods (How?)	Results
[13]	To assess the effect of music therapy on interactions among intergenerational age groups and the relationship to older adults’ psychosocial wellbeing.	Music therapy.	Questionnaires and analysis of the results of the participation of 21 children and 26 older adults in interventions based on singing, structured conversation, moving to music, and instrument playing.	Engaging in structured conversation and moving to music activities generated a more positive attitude towards aging and feeling more useful in the older adults.
[14]	To explore how ambient temperature variations influence mental health outcomes in older adults.	Ambient temperature.	National survey with data from over 2000 older adults, analysed for temperature-related impacts on mental health metrics.	Findings indicated a significant correlation between higher temperatures and elevated risks of mental health issues, especially anxiety and mood disorders.
[15]	To analyse how thermal, acoustic, visual, and indoor air quality (IAQ) factors influence stress levels in individuals with autism spectrum disorder (ASD).	Temperature, noise levels, lighting conditions, and air quality.	Survey-based study with 138 participants (caregivers and parents as proxy respondents), assessing perceived stress responses to different environmental conditions.	Acoustics was identified as the most stressful factor, followed by thermal, visual, and IAQ conditions. Higher autism severity correlated with greater sensitivity to environmental stimuli.
[16]	To examine how age, gender, co-morbidities, and autism severity influence stress responses to indoor environmental factors in individuals with autism spectrum disorder (ASD).	Temperature, noise levels, lighting conditions, air quality.	Survey-based study with 138 participants (parents and caregivers as proxy respondents), using statistical tests (ANOVA, Mann-Whitney) to analyse group differences.	Acoustics was the most stressful factor, with stronger reactions in individuals with severe autism. Gender and age differences were observed, with older individuals showing higher thermal and visual sensitivities.
[17]	To assess thermal sensation and skin temperature in older adults with and without dementia in care homes.	Indoor thermal comfort.	Observational study (69 older adults, 60–101 years). Thermal comfort assessed via self-reports, tympanic temperature, and infrared thermography.	Residents with dementia had lower extremity temperatures and reported feeling colder, despite similar ambient conditions.
[18]	To analyse how ambient noise levels and room acoustics impact communication and wellbeing in nursing homes for older adults with dementia.	Noise levels.	Acoustic measurements and predictive modelling in a nursing home in Flanders, Belgium. Analysis of pre- and post-intervention acoustic conditions after installing noise-absorbing materials.	Reducing reverberation time and optimizing sound absorption improved verbal communication and reduced acoustic stress for older adults with dementia.
[19]	To estimate the impact of traffic-related noise annoyance on health-related quality of life in a population-based study, according to gender.	Traffic-related noise.	Clinical study in 5021 participants, using multivariate regression methods, on the relationship between the effect of noise and chronic diseases.	A total of 13% of the sample reported a high level of traffic noise annoyance. Women were more likely to report high noise annoyance.
[20]	To examine the effects of season and weather on mood (valence and activation) and travel satisfaction.	Season and weather.	Data analysis of 562 commutes created by 363 randomly sampled people, using smartphones to report their mood state in their home before and directly after their daily travel.	Temperature leads to a more positive mood. Sunshine, despite making travel more relaxed, leads to a more negative mood for pedestrians and cyclists. Rain and snow result in a higher cognitive-assessed quality of travel.
[21]	To characterise personal and neighbourhood contextual influences on social isolation and loneliness among older adults.	Social isolation and loneliness.	Analysis of results from 124 interviews conducted with independently-dwelling persons with a mean age of 71, applying a convergent mixed-methods design and using quantitative and qualitative approaches.	Participants with a higher socioeconomic status, and those who lived closer to the city centre were less likely to report feeling socially isolated, while poor physical and mental health enhanced the feeling of isolation, especially among those living in low-density neighbourhoods.
[22]	To study the association between objectively obtained socioeconomic, physical, and social aspects of the neighbourhood in which persons live and the presence and severity of depressive and anxiety disorders.	Socioeconomic, physical, and social characteristics of a neighbourhood.	Analysis of outcomes of psychiatric interviews with 2980 participants, using multivariate regression techniques, with neighbourhood characteristics comprising a selection of socioeconomic, physical, and social factors.	Neighbourhood socioeconomic factors (low socioeconomic status, more social security beneficiaries, and more immigrants), physical factors (high levels of traffic noise), and social factors (lower social cohesion and less safety) were associated with the presence of depressive and anxiety disorders.
[23]	To develop an instrument to assess older adults’ perceptions of a set of environmental factors and to analyse their impact on their physical and psychological health, social relations, and environment.	Housing, facilities, residents, stench/noise and traffic.	Analysis of questionnaires (comprising 42 questions on ambient factors) conducted with 1031 individuals aged 65 or over, to determine the relationships between these factors and quality of life.	Characteristics of housing, residents, and nuisances impact on older persons’ quality of life. The other factors analysed showed a very limited effect on physical and mental wellbeing.
[24]	To assess the impact of music therapy interventions on reducing anxiety and depression in elderly patients with dementia.	Music therapy.	Randomized controlled trial with 150 elderly dementia patients; sessions measured for emotional responses and symptom changes.	Results showed that music therapy significantly reduced both anxiety and depression scores, providing a beneficial nonpharmacological option.
[25]	To explore how thermal environments influence comfort and autonomy in older adults with dementia, particularly regarding their control over temperature in home settings.	Indoor thermal comfort.	Semi-structured interviews with 5 older adults (aged 79–82) with early-stage dementia, combined with scenario simulation testing of various home thermal conditions.	Older adults with dementia prefer natural ventilation and passive heating (e.g., fireplaces, hot water bottles) over mechanical climate control. Autonomy over thermal settings enhances psychological comfort and engagement in daily activities.
[26]	To explore the impact of neighbourhood and home on health and cognitive decline in older adults.	Characteristics of household, neighbourhood, and social environment.	Analysis of the effect of characteristics of household, neighbourhood, and social environment on the wellbeing of 2206 older persons, using measurable objective criteria and experts’ opinions.	The household and social environment have a significant impact on cognitive functioning in older persons, with control being key to reduce future risks of cognitive decline.
[27]	To examine how exposure to residential green and blue spaces correlates with psychiatric disorder risks among middle-aged and older adults.	Residential green and blue spaces.	Analysis of UK Biobank data from over 5000 older adults, assessing green/blue space exposure against mental health outcomes.	Significant association found; greater exposure to these natural spaces was linked to lower incidences of psychiatric disorders, suggesting protective environmental benefits.
[28]	To examine the possible associations between daily weather conditions and thoughts, feelings, behaviours, and, by extension, life satisfaction.	Weather.	Evaluation, by means of questionnaires and statistical tests, of the behaviour and responses of 1 million United States citizens over a 5-year period.	The results show that weather does not reliably affect judgments of life satisfaction.
[29]	To investigate the possible effect of the presence of natural environments on subjective wellbeing.	Greenness and biodiversity.	Use of linear regression models to investigate the relationship between visualising green spaces, biodiversity, and blue spaces and subjective wellbeing in a sample of 4912 adults.	The existence of greenness and biodiversity is associated with subjective wellbeing. The relationship between the presence of private greenness and the sense of wellbeing is highlighted.
[30]	To determine the effects of music listening on cognition and acute confusion of older adults after hip or knee surgery.	Music therapy.	Analysis of the results of the NEECHAM Acute Confusion Scale and the Folstein Mini-Mental State Exam administered to 22 older adults after surgery and music therapy to measure their level of cognition and acute confusion.	The findings demonstrated that the music-listening group had higher levels of cognitive function and less confusion than those who did not listen to music.
[31]	To explore the association between night-time environmental noise and suicide death in adults.	Night-time noise.	Statistical analysis of the relationship between the incidence of suicide and the level of night-time noise (obtained from the National Noise Information System) in a sample of 155,492 adults (30,498 aged 55 years or over and 34,615 with mental illness)	With interquartile range increases in night-time noise, the hazard ratio for suicide death was significantly increased: 1.32 (95% CI: 1.02–1.70) for younger adults, 1.43 (95% CI: 1.01–2.02) for older adults, and 1.55 (95% CI: 1.10–2.19) for adults with mental illness.
[32]	To analyse the impact of noise on sleep quality according to age, gender, marital status, education, and body mass index.	Aircraft noise.	Use of logistic and linear regression models to assess data and objective parameters of sleep quality in 112 participants living in the vicinity of an airport over eight nights.	Increased aircraft noise events increased the time required for sleep onset (SOL) and the total wake time after sleep onset (WASO) and decreased sleep efficiency (SE), total sleep time (TST), and time in bed (TB) across all age groups.
[33]	To investigate the possible interaction between the presence of natural environments and environmental noise levels in urban settings on the psychological and physiological status of female adults.	Natural urban environments and noise levels.	Statistical analysis of the results of experiments with 83 adult women exposed to different natural urban environments and the measurement of psychological and physiological parameters.	Significant associations were found between the presence of natural environments, and to a lesser extent, noise levels, and psychological wellbeing, but not in the case of physiological parameters such as blood pressure.
[34]	To estimate the risk conferred by high ambient temperature on the health status of patients with psychosis, dementia, and substance misuse.	Hot weather.	Application of time-series regression analysis to data on 22,562 deaths over 9 years and daily weather information.	Patients with mental illness showed an overall increase in risk of death of 4.9% (95% CI 2.0–7.8) per 1 °C increase in temperature above the 93rd percentile of the annual temperature distribution.
[35]	To investigate the long-term cognitive effects of noise pollution exposure on older adults.	Noise pollution.	Longitudinal study with 1200 participants tracked over five years, focusing on cognitive assessments and environmental noise levels.	Consistent exposure to higher noise pollution was associated with accelerated cognitive decline, especially in memory retention and processing speed.
[36]	To assess the thermal comfort of older adults in nursing homes and its impact on their wellbeing.	Indoor temperature.	Field study in six nursing homes (322 residents, 187 staff). Long-term monitoring of indoor air temperature and humidity, plus thermal comfort surveys for both residents and staff.	Older adults preferred warmer temperatures (~0.9 °C higher than staff preferences) and wore more clothing. Some facilities failed to provide adequate thermal comfort, particularly in summer and winter.
[37]	To evaluate the impact of thermal comfort and HVAC design on older adults with dementia, focusing on their unique thermoregulatory needs.	Indoor temperature and air quality.	Literature review and qualitative research using semi-structured interviews with caregivers and professionals in dementia care. Includes analysis of previous case studies on HVAC implementation.	Older adults with dementia show higher sensitivity to temperature variations due to altered thermoregulation. Poor HVAC design can exacerbate behavioural issues. Smart climate control and passive design strategies improve both comfort and quality of life.
[38]	To investigate how thermal comfort and HVAC design impact the wellbeing of older adults with dementia, considering their altered thermoregulation and cognitive impairments.	Indoor thermal environment.	Mixed-method approach: Literature review and semi-structured interviews with caregivers and professionals in dementia care.	Older adults with dementia have altered temperature perception, leading to discomfort and behavioural changes. Poor HVAC design can exacerbate agitation, while adaptive climate control improves wellbeing.
[39]	To test sensitivity to environmental social stress as a mechanism of psychosis using Virtual Reality (VR) experiments.	Stress.	Analysis of results of experiments with 170 volunteer participants (55 with recent onset psychotic disorder, 20 at high risk for psychosis, 42 siblings of patients with psychosis, and 53 controls) where different levels of population density, ethnic density, and hostility were simulated.	Paranoia and subjective distress increased with the degree of social stress in the environment. Experimental evidence was provided to suggest that heightened sensitivity to environmental social stress may play an important role in the onset and course of psychosis.
[40]	To examine the relationship between the number of emergency department visits for “mental and psychosocial problems” and temperature and humidity in younger and older persons.	Temperature and humidity.	Analysis using Poisson regression and generalised additive models of the relationship between temperature and humidity (daily data over 12 years) and the number of emergency department visits for “mental and psychosocial problems” (347,552 visits).	The number of visits tended to increase with increasing mean temperature and higher-than-average humidity, although the effect of the latter was less significant in the group of older adults (aged 65 or over).
[41]	To investigate the relationship between ambient temperature and hospital admissions for schizophrenia by age group.	Temperature.	Analysis using regression models for the relationship between the number of hospital admissions for schizophrenia (over 9 years) and daily temperature data according to age groups (17,744 admissions, 737 of whom were aged 61 years or over).	A relationship was found between ambient temperature and admissions for schizophrenia across all age groups. A temperature of 28 °C (75th percentile) was associated with a 7% increase in admissions (95% confidence interval: 4e11%). Nonetheless, the oldest age group (61 years or over) was not the most affected.
[42]	To analyse the impact of social engagement on depressive symptoms in elderly individuals through a meta-analysis.	Social environment.	Meta-analysis of 25 studies encompassing a total of approximately 5000 older adults, examining social interaction frequency and depressive symptoms.	Higher levels of social engagement were strongly associated with reduced depressive symptoms, highlighting the importance of social interaction for mental health.
[43]	To evaluate the effect of music therapy on the symptom management and coping skills of patients receiving palliative care.	Music therapy.	Statistical analysis of results of surveys administered to 53 palliative care patients and 57 family members regarding experiments.	All patients reported that music therapy facilitated stress relief, relaxation, pain relief, spiritual support, emotional support, and a general feeling of wellbeing.
[44]	Investigate the impact of the built environment and care services on the quality of life of older adults in rural Chinese nursing homes.	Architectural design, medical care, and environmental factors like social environment, greenness, noise, and air quality.	Survey with 242 residents of rural nursing homes; statistical analysis of environmental and service-related factors; multiple regression analysis.	The study recommends enhancing ventilation, noise control, green spaces, accessibility, and social environment to improve older adults’ well-being.
[45]	To explore how social environment interactions contribute to empowerment and well-being in individuals living with dementia.	Social environment.	Qualitative study involving interviews with 40 dementia patients, focusing on experiences of social interaction and personal empowerment.	Positive social interactions enhanced feelings of empowerment and emotional well-being, demonstrating the supportive role of social environments in dementia care.

**Table 4:** Summary of the results of the selected studies.

Studied Factor	Mental Wellbeing		Physical Well-Being	
	Influential	Not Influential	Influential	Not Influential
Music (n = 4)	4 (100%)	0 (0%)	NA	NA
Noise (n = 11)	10 (100%)	0 (0%)	2 (100%)	0 (0%)
Weather (n = 13)	12 (92.31%)	1 (7.69%)	NA	NA
Social environment (n = 7)	7 (100%)	0 (0%)	2 (100%)	0 (0%)
Greenness (n = 4)	3 (100%)	0 (0%)	0 (0%)	1 (100%)
Stress (n = 1)	1 (100%)	0 (0%)	NA	NA
Lighting conditions (n = 2)	2 (100%)	0 (0%)	NA	NA
Air quality (n = 3)	3 (100%)	0 (0%)	NA	NA
